# A dose perturbation tool for robotic radiosurgery: Experimental validation and application to liver lesions

**DOI:** 10.1002/acm2.13766

**Published:** 2022-09-12

**Authors:** Ming Liu, Joanna E. Cygler, Kristopher Dennis, Eric Vandervoort

**Affiliations:** ^1^ Department of Medical Physics The Ottawa Hospital Cancer Center Ottawa Canada; ^2^ Department of Physics Carleton University Ottawa Canada; ^3^ Division of Medical Physics, Department of Radiology The University of Ottawa Ottawa Canada; ^4^ Division of Radiation Oncology The Ottawa Hospital and the University of Ottawa Ottawa Canada

**Keywords:** delivery errors, dose perturbation, liver, radiochromic film, stereotactic body radiation therapy

## Abstract

**Background:**

An analytical tool is empirically validated and used to assess the delivered dose to liver lesions accounting for different types of errors in robotic radiosurgery treatment.

**Material and methods:**

A tool is proposed to estimate the target doses taking into account the translation, rotation, and deformation of a target. Translational errors are modeled as a spatial convolution of the planned dose with a probability distribution function derived from treatment data. Rotations are modeled by rotating the target volume about the imaging isocenter. Target deformation is simulated as an isotropic target expansion or contraction based on changes in inter‐fiducial spacing. The estimated dose is validated using radiochromic film measurements in nine experimental conditions, including in‐phase and out‐of‐phase internal‐and‐external breathing motion patterns, with and without uncorrectable rotations, and for homogenous and heterogeneous phantoms. The measured dose is compared to the perturbed and planned doses using gamma analyses. This proposed tool is applied to assess the dose coverage for liver treatments using D99/Rx where D99 and Rx are the minimum target and prescription doses, respectively. These metrics are used to evaluate plan robustness to different magnitudes of rotational errors. Case studies are presented to illustrate how to improve plan robustness against delivery errors.

**Results:**

In the experimental validations, measured dose agrees with the estimated dose at the 2%/2 mm level. When accounting for translational and rotational tracking residual errors using this tool, approximately one‐fifth of targets are considered underdosed (D99/Rx < 1.0). If target expansion or contraction is modeled, approximately one‐third of targets are underdosed. The dose coverage can be improved if treatments are planned following proposed guidelines.

**Conclusion:**

The dose perturbation model can be used to assess dose delivery accuracy and investigate plan robustness to different types of errors.

## 1 INTRODUCTION

Treatment delivery errors due to target motion and deformation in radiation therapy may result in insufficient dose to targets and excessive dose to organs at risk (OARs). Several methods have been proposed to evaluate the dosimetric impact of delivery errors.^1^, ^2^, ^3^, ^4^, ^5^, ^6^, ^7^, ^8^ Lujan et al.^1^ proposed a convolution method to estimate the dose delivered to liver lesions subject to intrafractional motion. They convolved planned dose with an organ motion probability distribution function, based on a simple mathematical breathing model, expressed as a function of position rather than a function of time. In a second method, they recalculated time‐weighted average dose over 10 bins, by shifting the isocenter and beams to emulate target motion. The doses calculated using the two methods agreed within 2% in over 98% of the healthy liver volume. Similarly, using these two methods, Karlsson et al.^2^ found that the minimum target doses calculated using the two methods were within 2% for lung treatments when accounting for translational uncertainties (up to 10 mm). Ravkilde et al.^3^ proposed a fast dose reconstruction method which took into account the translational motion of targets by employing a pencil beam convolution algorithm. In their experiments, the dose was measured using a motion phantom containing two planar diode arrays. The phantom motion emulated the target motion observed in lung and prostate cancer patient treatments. The measured dose agreed with the reconstructed dose at the 3%/3 mm level. They also validated this method in phantom^9^ and planning^10^ studies for liver treatments.

To our knowledge, few studies have quantified the dosimetric effects of target rotations for stereotactic body radiation therapy (SBRT) liver treatments .^4^, ^5^ Shiinoki et al.^7^ assessed the dosimetric impact of errors in respiratory gated beam delivery with real time motion monitoring of implanted fiducials for lung tumors. They used a high precision robotic arm to translate and rotate the phantom based on the observed motions of patients during conventional gantry mounted linac beam delivery. Gafchromic films were embedded in the phantom and measured dose was compared for static and dynamic delivery. For 15 cases considered, they found that translational motion of lung tumors could be compensated for effectively, but agreement between the static and dynamic phantom dose at the 3%/2 mm level were significantly poorer when rotational motions (1.2°‐1.6° about different anatomical axes) were present. Alternatively, the dosimetric impact of target rotation can be assessed using observed setup errors for patients.^5^, ^11^, ^12^, ^13^, ^14^ For seven patients treated on a gantry based linac, Cao et al.^5^ recalculated the dose using CT image sets that were rotated by setup errors about the patient's superior–inferior axis. For three cases with setup errors >2.5°, the fraction of the PTV receiving >80% of the prescription dose was reduced by 7.5% ± 1.3%. For 24 patients treated using CyberKnife (Accuray Inc., Sunnyvale, CA), Chan et al.^4^ rotated and translated the planning target volume (PTV) contours by aggregate translational and rotational errors (<0.7° in any direction) binned and averaged across 10 breathing phases. They found that the median PTV dose was reduced by 1.1% for their patient cohort. In a previous study of liver SBRT treatments at our center,^12^ we found that rotational corrections were often larger than those observed by Chan et al. and exceeding what could be corrected by the CyberKnife system. We estimated that these uncorrected rotational errors alone could lead to 2.7% ± 5.8% reduction of the near minimum dose to targets using 5 mm margins.

In this work, we propose a dose perturbation tool to estimate the dose delivered to targets taking into account uncorrected target rotation, translational errors, and deformation. We first validate the perturbation model by comparing estimated dose with Gafchromic™ EBT3 (Ashland Inc., Covington, KY) film measurements^15^, ^16^, ^17^ under different conditions and for different motion patterns. Then we apply the tool to retrospectively assess the target doses for liver cancer treatments on CyberKnife.

## 2 METHODS AND MATERIALS

### 2.1 Principles of system operation and treatment data

The CyberKnife™ image‐guided stereotactic radiosurgery system employs a compact 6 MV linear accelerator mounted to a robot arm, and photon beams which can be shaped using a set of fixed‐diameter circular tungsten collimators (5 to 60 mm cones). The robot arm can change the beam directions to compensate for translations and rotations of the target during treatment. For treatments using the Synchrony respiratory motion compensation system,^18^ a correlation model is created based on the locations of internal fiducials (we typically use ≥3 platinum seeds implanted near the tumor) and external LED markers (placed on the patient surface). The model is either linear or curvilinear^19^, ^20^ with potentially different models in the inhale and exhale phases. The fiducial locations are obtained by orthogonal planar kilovoltage imaging (every 1‐2 min), and marker locations are measured using a camera array (every 40 ms). During treatment, the system predicts the tumor location using the correlation model and marker locations and corrects for the tumor motion in real‐time. With the Synchrony motion compensation system, patients breathe freely during treatment. Patients are treated supine immobilized using a custom Vac‐Lok™ with their arms at their sides. All treatment plans used fixed cones, with the sequential optimization with either the ray‐tracing^20^, ^21^ or Monte Carlo^22^ dose calculation algorithms depending on the proximity to the diaphragm/lung interface.

Different types of delivery errors associated with motion tracking are derived from treatment delivery log files. For each x‐ray image acquisition, a correlation error is recorded (typically <2 mm for our patient cohort) which is the difference between the predicted and measured target positions. A predictor error (usually <1 mm) is also reported every 40 ms, representing error due to system latency (115 ms).^23^ The end‐to‐end error (<1 mm in any direction^24^, ^25^) represents the overall geometric delivery accuracy of the system measured in a static phantom as part of routine CyberKnife quality assurance. These three types of errors are considered independent sources of translational tracking residual errors.^26^

Rotational corrections about the imaging isocenter are estimated by the system^27^ for every x‐ray image acquisition if certain criteria^12^, ^28^ are met for fiducial tracking. The magnitude of rotation that the CyberKnife system can correct depends on the tracking method, treatment couch, and the path set used for treatment. A path is a set of predefined positions, also referred to as nodes, where the robotic arm can move the linac target.^20^ For treatments using the Synchrony system, body path, and standard treatment couch, the upper limit of rotational corrections is ±1.5° in roll and pitch and ±3° in yaw. For each x‐ray image acquisition, we can calculate a signed rigid body error^12^ to describe target deformation, which is the average change in the pairwise inter‐fiducial distances, relative to the planning CT. The correction angles, which are yaw (*α*), pitch (*β*), and roll (*γ*), describe the target orientation during CT imaging relative to that at the acquisition of an x‐ray image. This frame of reference is right‐handed, defined using the patient “head first supine” orientation, with yaw, pitch, and roll rotations about the anterior (z+), left (y+), and inferior (x+) directions, respectively. The corrections for target rotations are applied in the following order: yaw, pitch, and roll.

In this study, all data were collected with the informed consent of patients as a part of a Research Ethics Board (protocol number 20160594‐01H) approved local clinical trial. This study includes 148 treatment courses for liver lesions (metastases/primaries: 70%/30%, age: 69 ± 11 years, M/F: 39%/61%). The Synchrony motion compensation system (delivery system v.9.0‐10.5; planning system v.3.5‐5.2) is employed for these treatments on CyberKnife. The prescription dose is between 42 to 60 Gy in three to six fractions. In a previous study examining 72 patients treated for liver lesions at our center,^12^ we found that 4 mm isotropic margins were sufficient to account for motion tracking, beam positioning, and rigid body errors in 95% of the population. For comparison, the target motion ranges observed in that study were 17.5 ± 5.3, 4.0 ± 1.9, and 7.5 ± 3.3 mm, respectively, for the superior–inferior, left–right, and anterior–posterior directions, respectively. At our center, a more conservative 5 to 7 mm isotropic PTV expansion from the gross tumor volume (GTV) is used in practice.

Treatment planning is done on an end‐expiration breath‐hold CT. The GTV is typically derived using an expiration breath‐hold MR images with Primovist^®^ contrast and/or a CT scan using iodine based intravenous contrast. We do not use a clinical target volume (CTV) margin for liver SBRT treatments following the approach adopted for lung SBRT trials (e.g., trial RTOG 0618^29^) by the Radiation Therapy Oncology Group (RTOG). The planned dose is usually calculated using ray‐tracing, but Monte Carlo calculation^21^ is employed if targets are close to the diaphragm.

### 2.2 Dose perturbation tool

**FIGURE 1:**
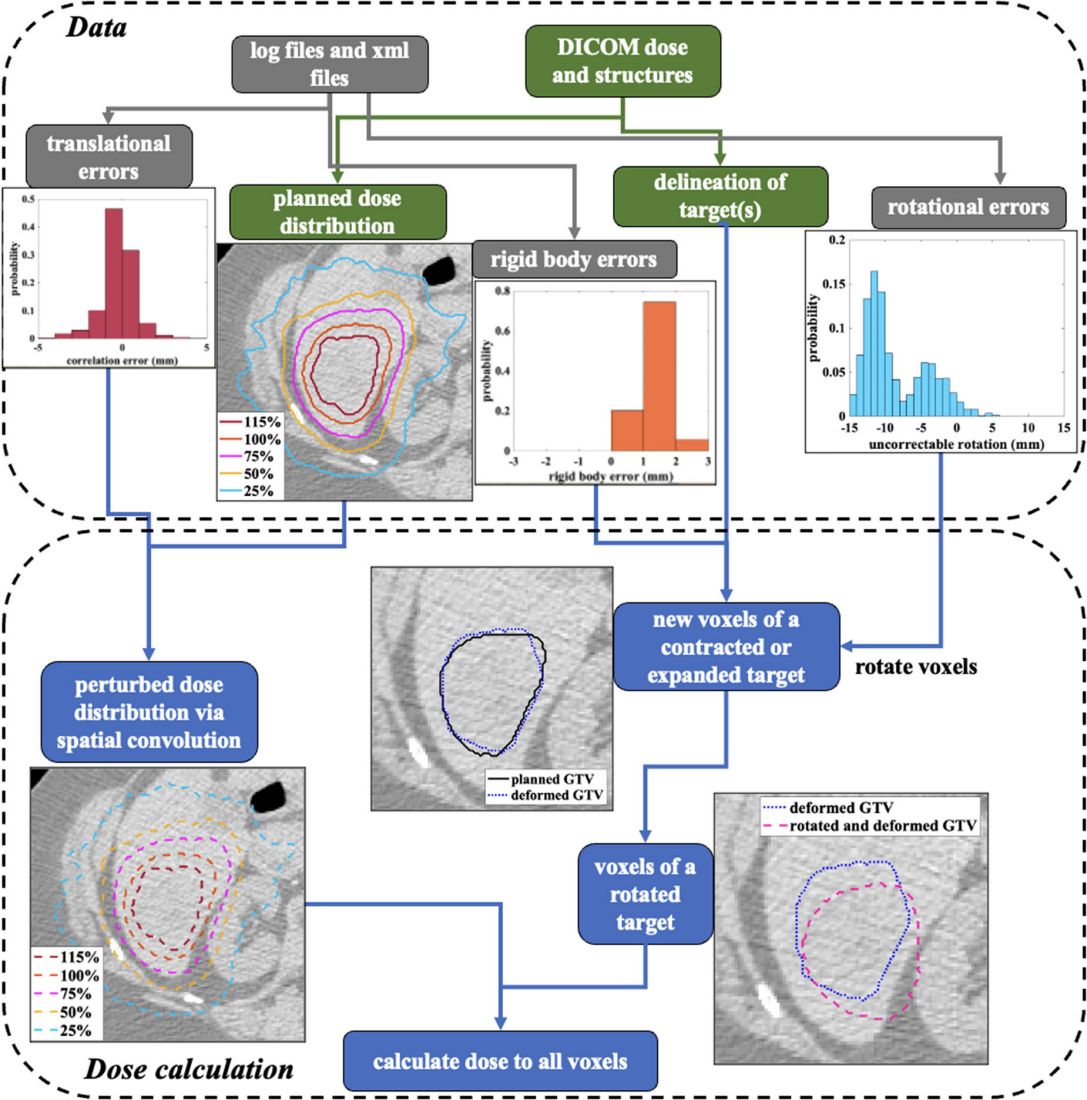
The flowchart illustrates the dosimetric perturbation tool. Three types of treatment delivery errors are considered in assessing the target dose: translational errors, uncorrectable target rotations, and target deformations (rigid body errors)

Figure 1 illustrates how the dose perturbation tool is implemented to account for translational errors, uncorrectable rotation, and target deformation. First, we convolve the planned dose with the probability density functions for correlation, predictor, and end‐to‐end errors which have been discretized to the dose‐matrix bin size. The end‐to‐end errors are obtained from quality assurance measurements made using a static phantom with fiducial tracking between 2015 and 2019. The correlation and predictor errors are derived from system logfiles extracted for each patient and filtered to only include data which was used for patient treatment (e.g., correlation errors which were discarded by radiation therapists are not included).

The target voxel positions after rotation are discretized using the nearest neighbor approximation, and the dose at each new position is determined using the post‐convolution dose. If a reported rotation can be corrected by the system, it is assumed to be zero in the dose perturbation model. We employ a simple model to account for target deformation based on changes in the inter‐fiducial spacing (referred to as rigid body error in the CyberKnife system) observed during treatment. Based on the fiducials’ locations determined from each x‐ray, we isotropically contract or expand the GTV contour depending on the average distance between the fiducials getting closer or further apart, respectively. The accumulated doses in each voxel are equally weighted by all the rigid body errors and rotational corrections observed during a patient's treatment. In‐house MATLAB (2018b, MathWorks Inc., Natick, MA) scripts are implemented for this tool.

### 2.3 Experimental validation

We validate the tool by comparing the estimated target doses (hereinafter referred to as the perturbed doses) with film measurements. Only rigid phantoms are used for validation. The agreement between the planned (or perturbed) and measured doses is quantified by the gamma pass rate using 2%/2 mm or stricter criteria. The fast implementation of the gamma analysis described by Wendling et al.^30^ was used for this study. For experiments using a motion phantom, the systematic shift between the planned (or perturbed) and measured doses is determined by comparing dose‐weighted centroids for voxels with doses >80% of the maximum planned dose (D_max_). The penumbral width (half of the difference between full widths at 80% and 20% maximum) is calculated for the dose profile along the motion direction centered at the location of the maximum planned dose (D_max_) in the film plane. The measured dose usually has a negligible component from x‐ray imaging. Typically, less than 50 x‐ray images are taken per experiment which would contribute =0.1% of the planned maximum dose as estimated by the CyberKnife treatment delivery system.^31^ For experiments in which more than 100 x‐ray images are taken, an imaging dose correction is estimated based on our previous experimental data^17^ and subtracted from the measured dose.

For film processing, we use an Epson (Epson, Nagano, Japan) 10000XL scanner (48‐bit color, 150 DPI) in transmission mode, and process data using the green color channel. We follow Devic et al.’s^32^ approach to linearize the dose–response curve and adjusted the exponent in the numerator from 2/3 to 4/5 to maximize the R^2^ of the fit function. Dose rescaling is performed following Lewis et al.^33^ Three phantoms, described in the next section, are employed to validate the model, each having more than three fiducials embedded for accurate rotation tracking.^4^, ^34^ With the same film processing methods, we have previously estimated that the one sigma uncertainty for a single film measurement is approximately 2.3%.^17^ At least two films are therefore used in every experiment to reduce the dosimetric uncertainty of measurements.^35^, ^36^ Holes are punched in each film to rigidly fix it to the phantom using a minimum of two high density pins which are identifiable in the high‐resolution (⩽1 mm maximum voxel length) CT images acquired. The holes are visible in the 150 DPI scanned images. We estimate that the spatial uncertainty is better than 1 mm for film measurement localization with respect to the planning system predicted dose. Three measurement scenarios are described below in order of increasing complexity in terms of phantom material composition and motion patterns.

**FIGURE 2:**
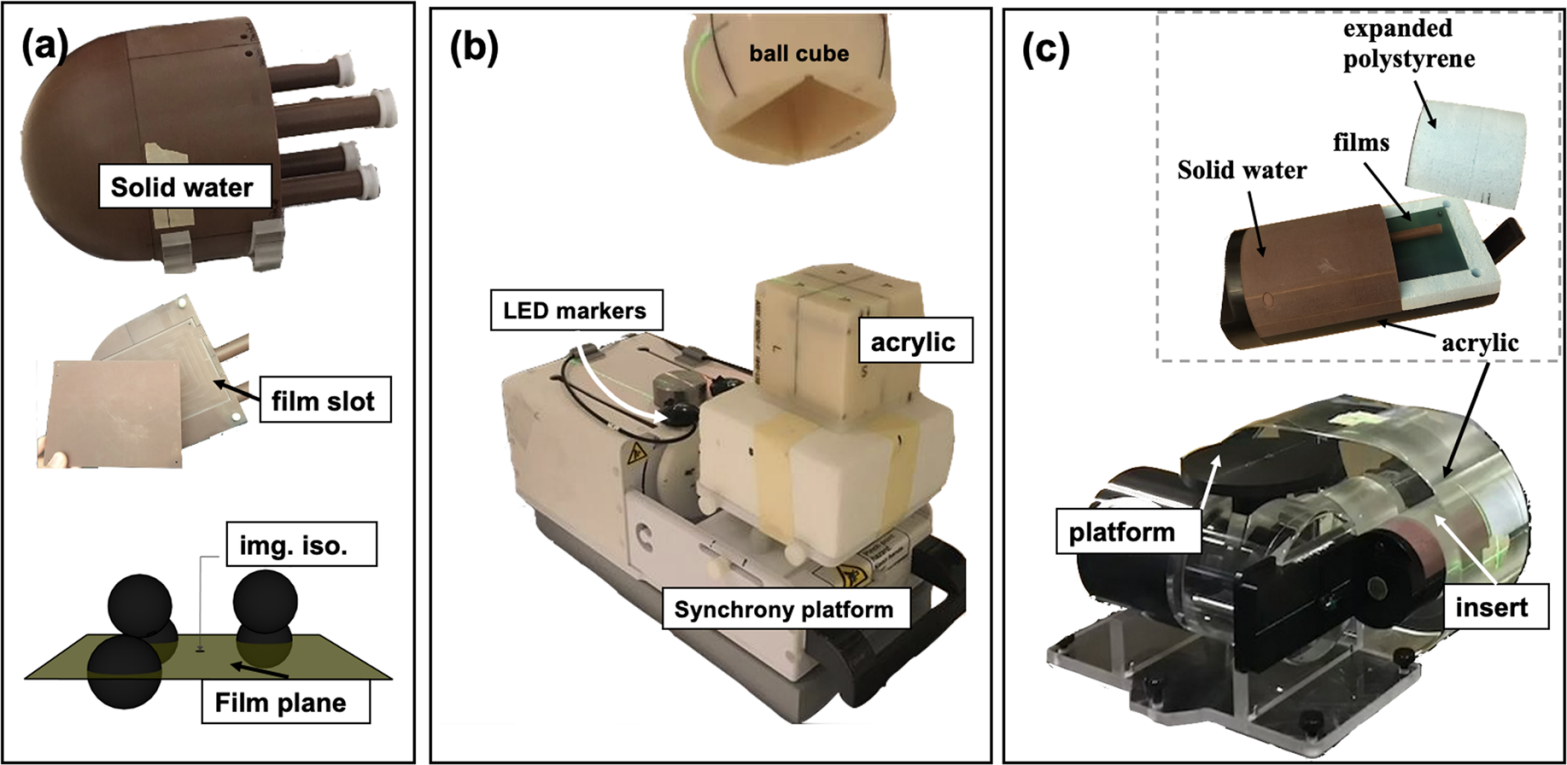
(a) A bullet shaped phantom made primarily of Solid Water™ with a slot for a stack of four films which were oriented in the sagittal plane. The lower diagram illustrates where the different beam isocenters are placed relative to the film plane. The imaging isocenter (img. iso.) is also shown. (b) The ball‐cube phantom and synchrony motion platform are provided by Accuray to perform quality assurance of the Synchrony respiratory motion compensation system. Two custom cut films are centered within the phantom and placed orthogonally in transverse and sagittal planes. The Synchrony platform moves the phantom (horizontally) and LED markers (vertically) in perpendicular directions with periodic motion and a variable phase shift. (c) A customized cylindrical insert designed for a Quasar™ respiratory motion phantom. The Quasar motor drives the cylinder using a user input motion trace to emulate internal breathing motion in the thorax or abdomen. A platform simultaneously moves vertically to emulate the breathing motion of the chest wall

The first scenario is used to enhance the dosimetric impact of uncorrected rotational errors. For this test a bullet‐shaped phantom (see Figure 2a) was used, meant to emulate a head‐like geometry, made primarily of homogeneous water‐equivalent material (Solid Water™). Four films (10.2 × 12.7 cm^2^) are placed in a sagittal plane of the phantom. The planned delivery employs isocentric delivery with the 10 mm collimator with a maximum planned dose (D_max_) of 10 Gy. The phantom remains *static* during beam delivery. As shown in the diagram in Figure 2a, five beam isocenters were placed with the three isocenters within the film plane, and two beam isocenters were laterally offset (5 mm) from the film plane to increase sensitivity to uncorrected rotations. The rotational offset for the phantom is 3.1°/−2.7°/3.1° for yaw/pitch/roll, greater than what could be corrected by the system.

The second scenario assesses the dosimetric impact of translational errors for motion tracking with simple out‐of‐phase internal and external motions. These tests were performed using the phantom and motion platform provided by Accuray for end‐to‐end quality assurance of the Synchrony system. A motor drives the phantom on a platform (see Figure 2b) with periodic motion in the superior–inferior direction with 20° or 30° out‐of‐phase vertical motion of the LED markers. The phantom is referred to as the ball‐cube phantom (see Figure 2b) and is primarily made of homogeneous plastic materials. It allows two films (6.3 × 6.3 cm^2^) to be placed orthogonally in the transverse and sagittal planes.^37^ The planned delivery is isocentric using the 25 mm collimator with a maximum planned dose (D_max_) of 10 Gy to the center of the films.

The third scenario assesses the combined dosimetric effect of rotational offsets and translational motion tracking errors using more realistic breathing motion (see Figure 2c). To emulate more complex motion, we employed a Quasar™ (Modus QA Inc., London, ON, Canada) motion phantom. A motor drives a cylindrical insert in one direction following motion patterns which can be input by the user. The motion waveform was derived from system logfiles files for a liver cancer patient treated at our center exhibiting a complex breathing pattern. The motor also drives a platform in the vertical direction with the same motion pattern but with a reduced amplitude. We place three LED markers on the motion platform to emulate in‐phase breathing motion of the chest wall. To create out‐of‐phase motion, the same motion pattern is run on two Quasar phantoms with a phase shift, with the LED markers on one Quasar and the insert containing the film and fiducials in the other. The same waveform is used for all six experiments. We estimate the phase shift for the out‐of‐phase experiments by fitting an ellipse to the fiducial position on y axis and marker displacement on x axis for a running window of fifteen data points. An average phase shift between the phantom and marker motions is calculated for each experiment.

As shown in Figure 2c, part of the cylinder consists of low density expanded polystyrene (in blue), with another section of Solid Water (in brown) to emulate the boundary between diaphragm and lung and a base made of acrylic (in black). For each experiment, two films (7.4 × 15.6 cm^2^) are positioned in the cylinder for each experiment. Two non‐isocentric plans are used: one with high dose (>50% D_max_) near the media boundary (denoted as “close‐to‐lung”), and high dose well inside the Solid Water (denoted as “far‐to‐lung”). In two of these experiments, the phantom is set up with a correctable (2°) and uncorrectable (4°) rotational offset in the yaw direction. They are labelled as Far2Lung‐4 and Far2Lung‐3, respectively, in Table 1.

**TABLE 1:** Parameters and results of the experiments employing a motion phantom to validate the dose perturbation model. In two of the experiments, the motion phantom is positioned with a rotational offset. The agreement between the planned (or perturbed) and measured doses is quantified by the gamma pass rate using 2%/2 mm or stricter criteria

Phantom	Label	Phase shift (degrees)	Correlation error (mm)	SD of predictor errors (mm)	Rotational offset (degrees)	Gamma criteria	*γ*_0_	*γ*_1_	Shift∥ (mm)	Shift⊥ (mm)	Penumbra width (mm)		
											Planned	Perturbed	Measured
Synchrony motion phantom + ball cube insert	Ball20	20	0.0 ± 0.8	0.2	N/A	1%/1 mm	97.4	98.1	−0.4	−0.5	5	6	6
						2%/2 mm	100.0	100.0					
	Ball30	30	−0.2 ± 0.7	0.2		1%/1 mm	95.0	96.7	0.3	0.2	6	7	7
						2%/2 mm	100.0	100.0					
Quasar motion phantom + liver insert	Far2Lung‐1	N/A	0.0 ± 0.3	0.4	N/A	2%/2 mm	95.4	96.0	0.2	0.4	11	12	12
						2%/1 mm	92.1	93.5					
	Close2Lung‐1		−0.0 ± 0.2	0.4		2%/2 mm	97.1	98.2	0.6	0.3	16	16	17
						2%/1 mm	85.0	94.0					
	Far2lung‐2	33^†^	−0.5 ± 2.1	0.4	N/A	2%/2 mm	93.2	98.8	2.0	1.6	12	13	14
						2%/1 mm	72.0	87.0					
	Close2Lung‐2	17^†^	0.1 ± 0.7	0.3		2%/2 mm	95.3	97.6	0.8	0.5	16	16	17
						2%/1 mm	80.0	91.5					
	Far2Lung‐3	49^†^	−0.6 ± 2.2	0.6	4.0	2%/2 mm	76.9	99.9	2.2	0.9	12	13	13
						2%/1 mm	63.0	96.8					
	Far2Lung‐4	43^†^	−0.3 ± 2.1	0.6	2.0^‡^	2%/2 mm	99.1	99.6	0.3	0.1	12	13	12
						2%/1 mm	95.6	98.1					

Notations: *γ*_0,_ gamma pass rate (%) comparing the planned and measured dose distributions (all non‐zero voxels); *γ*_1_, gamma pass rate (%) comparing the perturbed and measured dose distributions (all non‐zero voxels); shift∥, systematic shift of dose parallel to phantom motion direction comparing the planned and measured dose distributions; shift⊥, systematic shift of dose perpendicular to phantom motion direction comparing the perturbed and measured dose distributions.

^†^ The average phase shift estimated by fitting an ellipse to a 15‐data point running window to the fiducial versus marker positions.

^‡^ A rotational offset that can be corrected by the system (equivalent to zero degrees for the resulting rotational offset).

### 2.4 Patient dose assessments

For our patient cohort, 70 out of 148 treatment courses had rotational corrections that could be accurately calculated and tracked by the system. For 70 treatment courses that had target rotations accurately calculated by the system, we estimated treated D99/Rx for GTVs accounting for delivery errors using the dose perturbation tool (Rx represents the prescription dose) and compare it with planned D99/Rx. A target is considered underdosed if the treated D99/Rx < 1. Since the expansion/contraction model may oversimplify organ deformation, we report the treated D99/Rx values considering and neglecting the rigid body errors, where the GTVs are modelled as deformed and rigid targets, respectively.

Many of these 70 treatment courses had more than one target (97 in total). We conduct a two‐sample t test at a significance level of 5% for these courses with at least one underdosed *rigid* targets (22 targets with treated D99/Rx < 1) to identify parameters related to dose coverage. The parameters include Rx, planned D99/Rx, target volume, target centroid‐to‐imaging isocenter distance, target rotation (mean and standard deviation), and translational tracking error (mean and standard deviation).

Since many patients did not have target rotation calculated by the system, the dosimetric impacts due to target rotations remain unknown for their treatments. We therefore retrospectively evaluate the robustness of treatment plans to uncorrectable rotations based on treated D99/Rx by varying the simulated angles of rotation about each axis (roll and pitch: ±1.5°, ±3.0°, ±4.5°, yaw: ±3.0°, ±4.0°, ±5.0°) and modeling the impact of translational tracking errors for all 148 plans (192 targets).

## 3 RESULTS

### 3.1 Experimental validation

**FIGURE 3:**
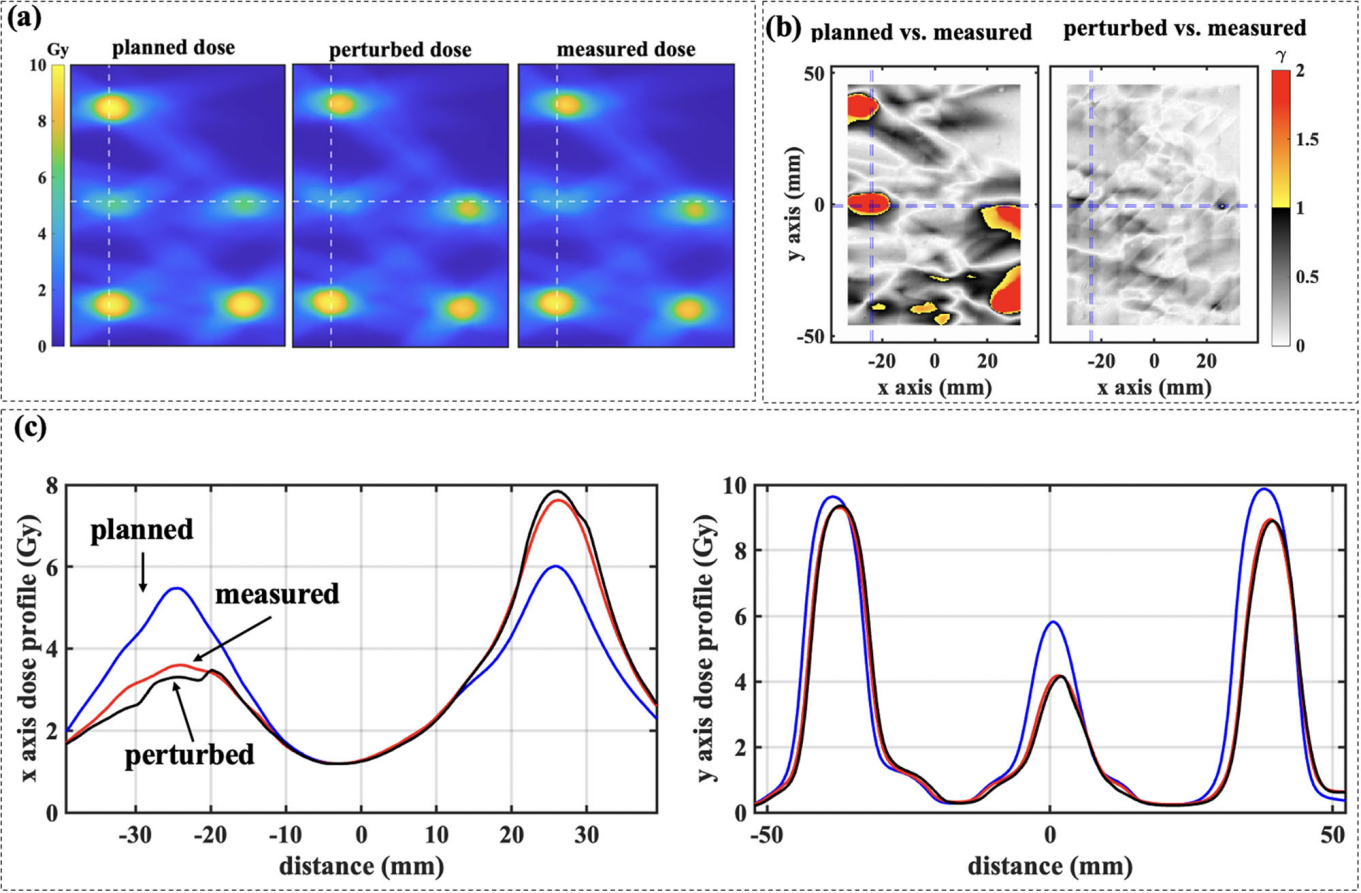
(a) The planned, perturbed, and measured dose of the film plane of a bullet‐shaped phantom that is placed with a rotational offset. (b) Two‐dimensional gamma analyses using 2%/2 mm criteria on planned and measured dose distributions (*γ*_0_ = 90.1%), and perturbed and measured dose distributions (*γ*_1_ = 100.0%). (c) The dose profiles are along the dashed lines in (a) and (b) for the planned, perturbed, and measured dose distributions

For the first scenario, Figure 3a illustrates the planned, perturbed, and measured doses of the film plane with uncorrectable rotational offsets and multiple targets. The gamma analyses between the planned and measured, and between the perturbed and the measured doses are illustrated in Figure 3b. The maximum difference between the planned and measured doses is as large as ±30% with a gamma pass rate (denoted as *γ*_0_ for planned vs. measured doses) of 90.1% using 2%/2 mm criteria (see dose profiles in Figure 3c). The gamma pass rate (denoted as *γ*_1_ for perturbed vs. measured) is increased to 100.0% because the dose difference between the perturbed and measured doses is significantly reduced.

Parameters and results for the Synchrony motion phantom and ball‐cube insert are listed in Table 1. The experiments labeled Ball20 and Ball30 employ 20° and 30° out‐of‐phase motion with no rotational offsets. The dose broadening effect is also minimal with measured penumbral width increasing by about 1 mm compared to planning. The penumbral widths of the perturbed dose are consistent with the measurements. Using 1%/1 mm criteria, a small increase in *γ*_1_ is observed as compared to *γ*_0_ for both experiments (see Table 1). Most gamma failures for *γ*_0_ are near the dose fall‐off region along the motion direction.

**FIGURE 4:**
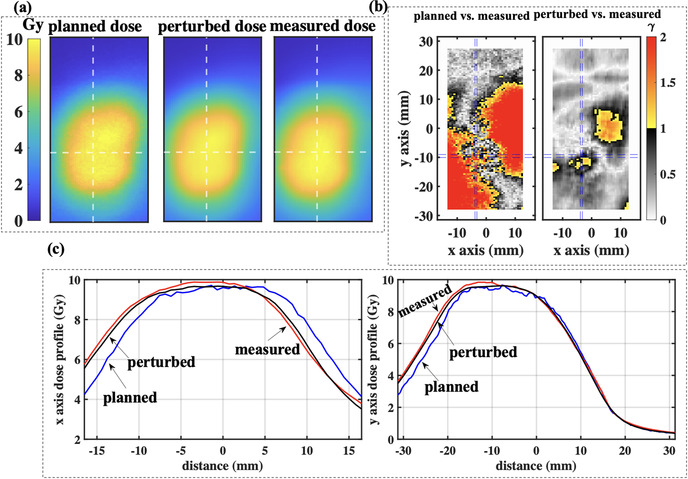
(a) The planned, perturbed, and measured dose in the film plane of a cylindrical phantom that is placed with an uncorrectable rotational offset. (b) Two‐dimensional gamma analyses using 2%/1 mm criteria on planned and measured dose distributions (*γ*_0_ = 63.0%), and perturbed and measured dose distributions (*γ*_1_ = 96.8%). (c) The dose profiles along the dashed lines in (a) and (b) for the planned, perturbed, and measured dose distributions

Results and parameters for the third scenario experiments are also shown in Table 1 for the Quasar motion phantom with the liver film insert. These experiments use more complex patient derived motion, with and without out‐of‐phase motion and rotations and in different proximity to low density media. Both the difference in the penumbral width and the systematic shift comparing the planned and measured doses are in most cases less than 2 mm. In all cases, *γ*_1_ is greater than *γ*_0_ indicating that gamma pass rates are improved when delivery errors are considered, particularly for the 2%/1 mm criteria. Figure 4a–c illustrates the planned, perturbed, and measured doses for experiment Far‐to‐Lung‐3. Using 2%/1 mm criteria, *γ*_0_ for experiments Far‐to‐Lung‐3 (rotation uncorrected) and Far‐to‐Lung‐4 (rotation corrected) are 63.0% and 95.6%, respectively, highlighting the discrepancy between planned and delivered dose when rotations are not corrected. In contrast, *γ*_1_ is greater than 96% for both experiments indicating that the dose perturbation model accurately predicts the impact of delivery errors.

### 3.2 Patient dose assessments

**FIGURE 5:**
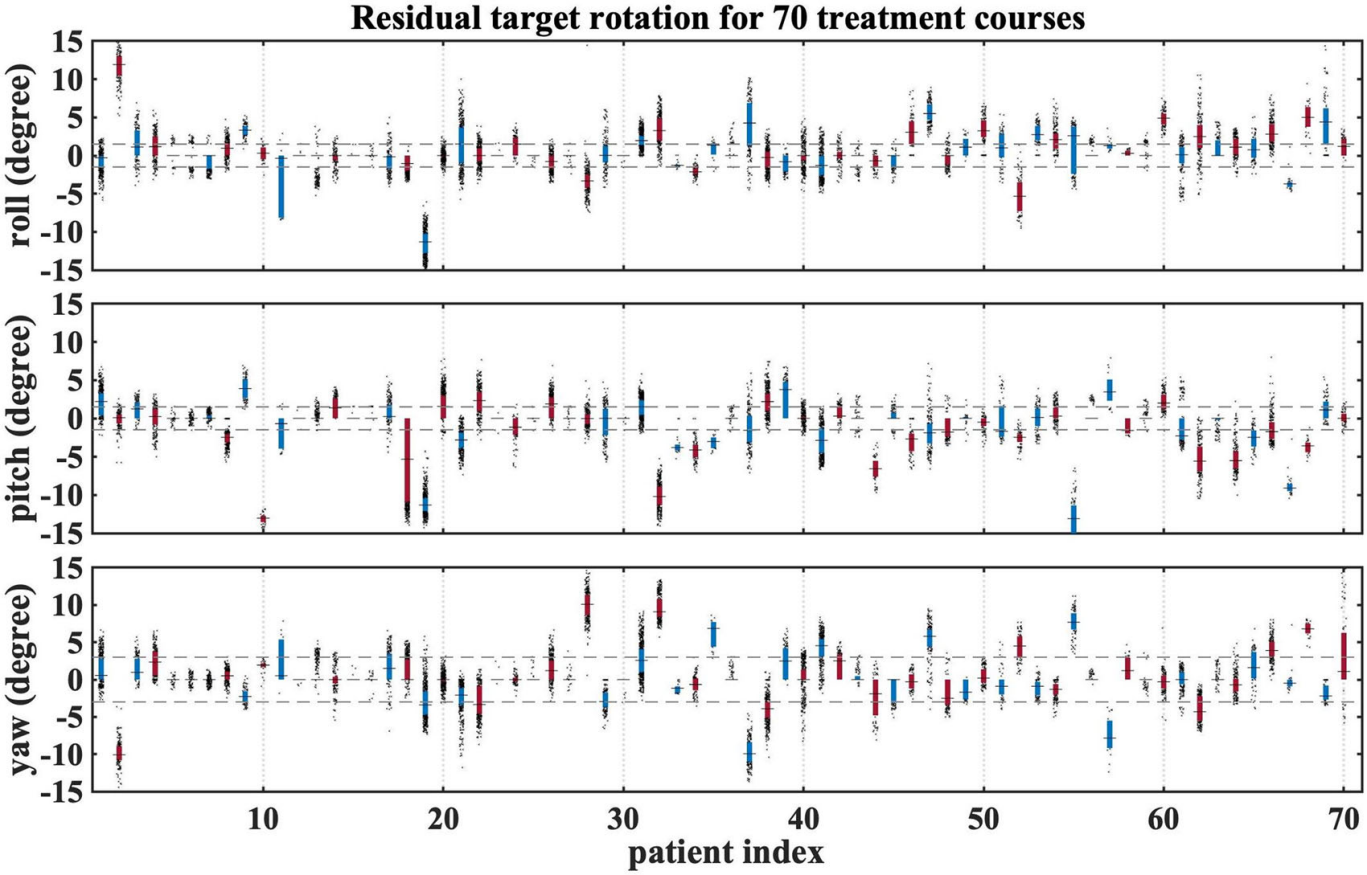
Target rotations in roll, pitch, and yaw are illustrated for 70 treatment courses (from left to right: treatment time in chronological order). Red and blue symbols alternate for different patients. For these patients, target rotations were tracked during treatment and met the criteria for accurate calculation of rotations. The horizontal dashed lines indicate the largest magnitude of corrections the system can apply. Each box spans from the first to the third quartile, and the horizontal solid line indicates the median. Data beyond the box are shown as black dots

**FIGURE 6:**
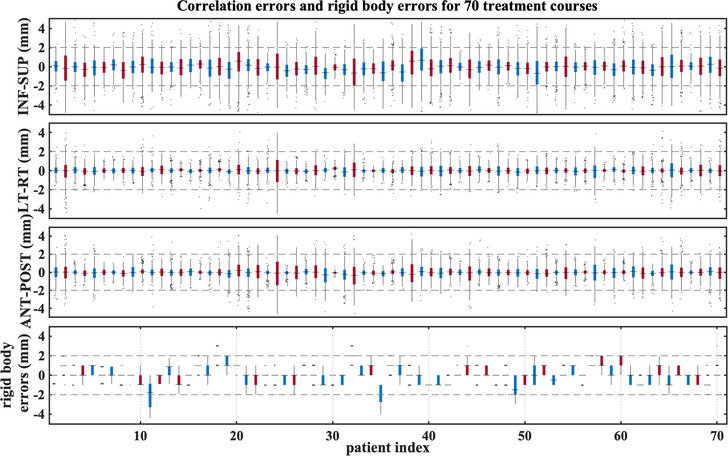
Correlation errors and rigid body errors are illustrated for 70 treatment courses (from left to right: treatment time in chronological order). Red and blue symbols alternate for different patients. The horizontal reference lines indicate errors of ±2 mm. Each box spans from the first to the third quartile, the vertical line represents the first to the 99th percentile of data, and the horizontal solid line inside the box indicates the median

**FIGURE 7:**
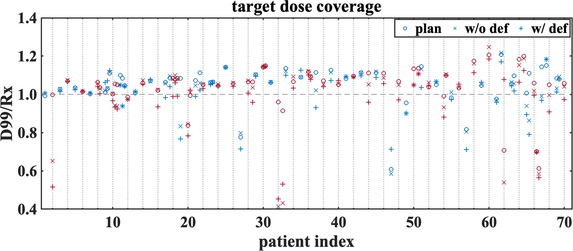
The planned and treated (with and without rigid body errors) D99/Rx are illustrated for 70 patients (97 targets). For treated D99/Rx, the translational and rotational errors are taken into account for rigid targets (labelled as w/o def). In addition to these two types of errors, target deformation is also considered (labelled as w/ def). Red and blue symbols alternate for different patients

For 70 treatment plans (97 targets), the implanted fiducials meet the criteria for accurately calculating rotations. Figures 5 and 6 show some of the inputs (residual uncorrected rotational errors, correlation errors, and rigid body errors) to the perturbation model for these treatment courses. Among them, 29 have an average rotational offset >3° in at least one of the elemental rotations, and 13 have the rotations corrected for more than 70% of the time. The impacts on D99/Rx considering different types of delivery errors are shown in Figure 7. In the original treatment plan, 18% of targets were already underdosed (planned D99/Rx < 1.0) indicating that coverage was compromised to spare nearby OARs. For rigid targets, 23% of targets are underdosed (treated D99/Rx < 1.0), while 35% of deformed targets are underdosed. We observe a change of more than 5%‐points in D99/Rx for 29% of GTVs when comparing rigid and deformed targets.

**FIGURE 8:**
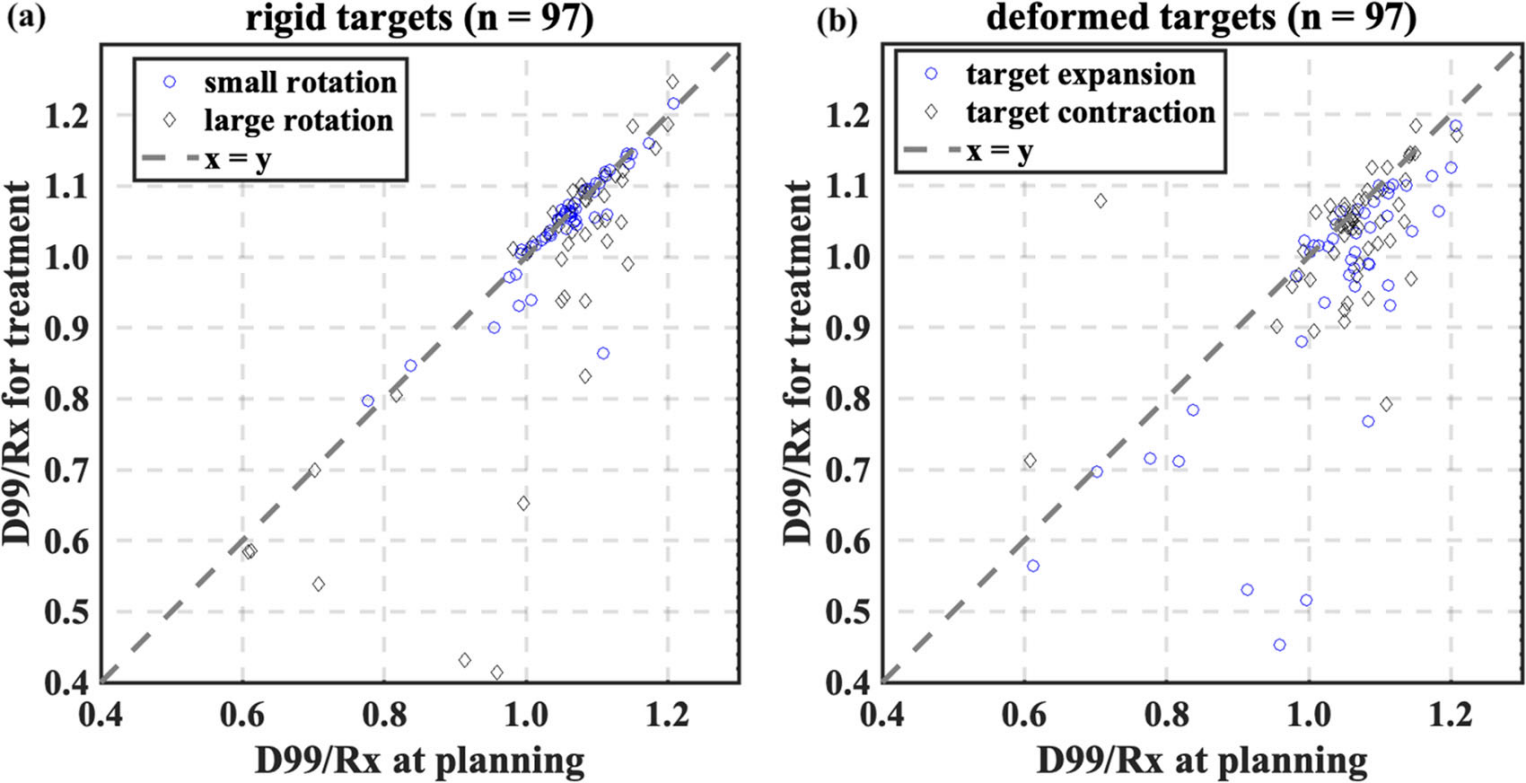
(a) The relationship between treated and planned D99/Rx is shown for 70 plans when target rotation and translational residual errors are considered. Data in the grey area indicate that targets receive less dose during treatment than planned. In (a), diamonds indicate treatments with average rotation error >3°, and circles represent the other treatments. (b) The relationship between treated and planned D99/Rx for 70 plans when target rotation, translational residual errors, and rigid body errors are considered. In (b), the circles indicate treatments with target expansion (the average rigid body errors > zero), and diamonds represent target contraction

The relationship between planned and treated D99/Rx for rigid and deformed targets is shown in Figure 8. In Figure 8a, diamonds indicate targets with the average rotation >3°, and in Figure 8b they indicate target contraction. For the rigid targets, we perform a two‐sample *t*‐test for various parameters in underdosed and non‐underdosed targets. The statistical test results are shown in Table 2. The planned D99/Rx, Rx, target‐to‐isocenter distances, the standard deviation of correlation errors, and rotational offsets in pitch (along the patient's left–right axis) are significantly different (*p* < 0.05) for underdosed versus non‐underdosed targets. We also perform a two‐sample *t*‐test for treatments with both a large rotational offset (>3°) and target centroid >2 cm away from the imaging isocenter (*n* = 41) compared to the remaining patients (*n* = 56). For these two groups, a statistically significant difference (*p* < 0.008 and 0.002, respectively) is found in the treated D99/Rx and the ∆D99/Rx (the difference between the planned and treated D99/Rx). In assessing the robustness of treatment plans to delivery errors for all 148 treatment plans, when simulated target rotation ≤3°, 23%, and 28% of targets are underdosed for rigid and deformed targets, respectively.

**TABLE 2:** Results of two‐sample two‐tailed *t*‐tests at 5% significance level for several potentially relevant quantities for underdosed^a^ and non‐underdosed^b^ targets considering translational and rotational tracking residual errors (but neglecting rigid body errors) for 70 patients (97 targets) with rotations tracked by the system

**Quantity compared**	**Underdosed targets** **^a^** **(*N* = 22)**	**Non‐underdosed targets** **^b^** **(*N* = 75)**	***p*‐value** **^c^**
Rx (Gy)	45.8 ± 4.0	48.3 ± 4.2	**0.03**
Planned D99/Rx	0.93 ± 0.16	1.08 ± 0.05	**<0.001**
Target volume (cc)	34.5 ± 70.3	33.2 ± 51.8	0.9
Target centroid‐to‐isocenter distance (cm)	5.6 ± 2.7	4.4 ± 1.9	**0.03**
Average roll angle (deg)	1.2 ± 4.2	0.4 ± 1.9	0.2
Average pitch angle (deg)	−3.7 ± 5.0	−1.0 ± 3.1	**0.003**
Average yaw angle (deg)	1.0 ± 4.7	0.5 ± 2.8	0.5
SD of roll angles (deg)	1.6 ± 0.9	1.4 ± 0.9	0.5
SD of pitch angles (deg)	1.5 ± 0.7	1.4 ± 1.0	0.9
SD of yaw angles (deg)	1.5 ± 0.7	1.4 ± 0.8	0.5
Average INF/SUP correlation error (mm)	−0.1 ± 0.2	−0.1 ± 0.2	0.8
Average LT/RT correlation error (mm)	0.0 ± 0.1	0.0 ± 0.1	0.3
Average ANT/POST correlation error (mm)	0.0 ± 0.1	0.0 ± 0.1	0.7
SD of INF/SUP correlation errors (mm)	1.4 ± 0.4	1.1 ± 0.3	**0.007**
SD of LT/RT correlation errors (mm)	0.7 ± 0.3	0.5 ± 0.2	**<0.001**
SD of ANT/POST correlation errors (mm)	0.8 ± 0.3	0.7 ± 0.3	**0.02**

^a^ “Underdosed” defined as treated D99/Rx < 1.

^b^ “Non‐underdosed” defined as treated D99/Rx ≥1.

^c^ Bold values denote statistical significance at *p* < 0.05 level.

**FIGURE 9:**
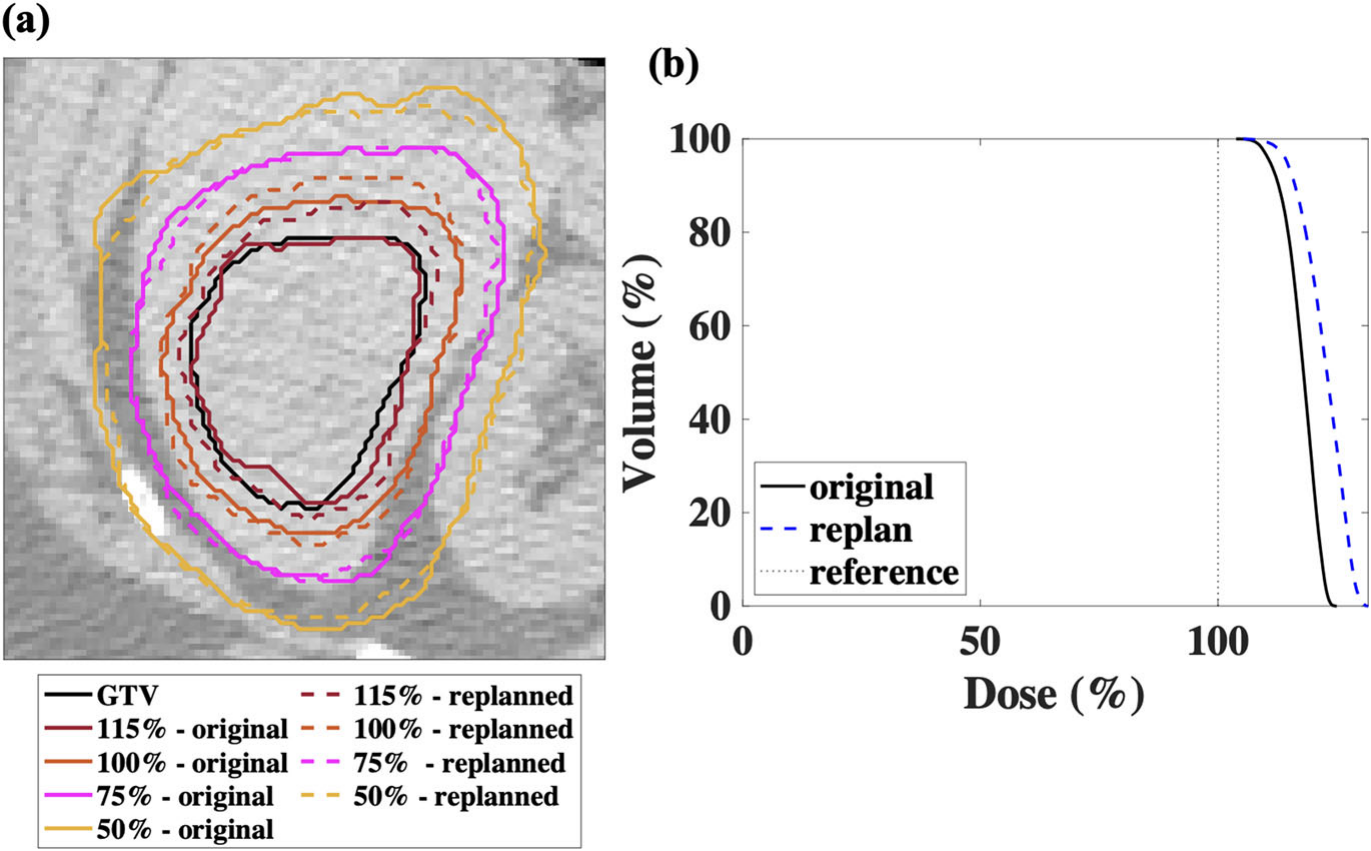
(a) A gross tumor volume (GTV) and the isodose lines relative to the prescription dose (48 Gy) for the original and replanned doses distributions on a transverse CT slice. (b) The dose volume histograms (DVHs) for the GTV for the original and replanned dose distributions

Based on the results of statistical tests above, treatments can be made more robust to delivery errors by having: (1) higher dose planned in the central part of GTVs, (2) reducing the target‐to‐imaging isocenter distance at planning, and (3) repositioning the patient to reduce the average rotational offset during patient setup. In Table 3, we illustrate the improvements in treated target coverage which can be made by making the first two changes for two representative underdosed cases. Figure 9 shows the isodose lines on a transverse CT slice and the dose volume histograms (DVHs) for the original and the new dose distributions for case 1.

**TABLE 3:** The near‐minimum dose (D99) to target(s) considering different delivery errors and for the original plan are compared for the two case studies. For each case we place the imaging isocenter close to the center of the target using the planned dose distribution. For each of them, a new plan is generated with higher dose delivered to the GTV and the imaging isocenter is placed as close as possible (within 3 cm) of the target centroids

**Details of the original plan**	**Changes in the simulation**	**Planned D99/Rx**	**Treated D99/Rx rigid target(s)**	**Treated D99/Rx deformed target(s)**
Case 1: Single target (28 cc), 4.5 cm between target centroid and imaging isocenter, large rotations (average target rotation: −3.0°, −11.1°, −11.4° for yaw, pitch, and roll, respectively).	Original plan, with original imaging isocenter	1.08	0.83	0.77
	Original plan, with imaging isocenter close to target centroid		1.06	0.99
	Hotter plan, with the imaging isocenter close to target centroid	1.11	1.09	1.00
Case 2: Two small targets (3 and 1 cc), treated in the same plan, each > 10 cm away from the imaging isocenter, with relatively small but uncorrectable rotations (average target rotation: 1.7°, −2.5°, 0.9° for yaw, pitch, and roll, respectively).	Original plan, with original imaging isocenter	1.01 and 1.11	0.94 and 0.86	0.89 and 0.79
	Original planned beams, but separated into two plans one for each target, with the imaging isocenter close to each target centroid		1.01 and 1.10	1.01 and 1.08
	Hotter plans, separate plans for each target, with the imaging isocenter close to each target centroid	1.05 and 1.19	1.05 and 1.18	1.04 and 1.15

## 4 DISCUSSION

### 4.1 Experimental validation

We first validate our proposed dose perturbation tool for treatments with uncorrectable rotations in a static Solid Water phantom. Then we evaluate the model for simple target motion with out‐of‐phase internal/external breathing motion using isocentric delivery in the homogenous ball‐cube phantom. The experiments using the Quasar insert demonstrate the model can be used to predict the impact of delivery errors on dose delivered for more complex clinical situations. These include employing patient‐derived breathing waveforms, in‐phase and out‐of‐phase motion, targets close to the lung–liver interface and with motion tracking errors combined with uncorrectable rotations. For close‐to‐lung experiments, the gamma failures are mostly in the penumbra of the measured dose (lower dose region) and near the media boundary. In contrast, for far‐to‐lung experiments, most gamma failures are in the high‐dose area. In our study, the dose blurring does not include the effects of individual beams intersecting and overlapping in high‐dose regions around the periphery of targets for non‐isocentric delivery. During the delivery of individual beams, errors might be of a different magnitude and sign, which could lead to changes in high‐dose regions not predictable by our model. It is also worth noting that the perturbation model presented does not account for changes in attenuation as beams pass through different thicknesses of tissue due to breathing motion or rotational offsets. We have previously investigated attenuation effects for this phantom^17^ and for three patients with large rotations.^12^ For the phantom, we observed changes between 1% and 3% in a high dose region in the extreme phases of respiration. For patients, we observed lower gamma pass rates if attenuation effects associated with rotations are neglected (minimum 91% pass rate for 2%/2 mm 3D gamma criteria, for voxels with >50% of the maximum planned dose). However, our experiments using a moving and rotated phantoms show good agreement between perturbed and measured doses (penumbral widths within 1 mm and *γ*_1_ > 96% using 2%/2 mm criteria) suggesting the impact of these effects are small.

In the experimental validations using dynamic phantoms, the systematic shift in the motion direction between film and the perturbed dose is reduced relative to the planned dose for all cases considered. For patients, Figure 6 shows that the signs of the correlation errors tend to cancel across a full treatment course with systematic errors close to zero. Dose broadening effects (due to the distribution of motion tracking errors) and uncorrectable rotations are important features to correctly predict the dosimetric impact of delivery errors for this system. For all experiments, translational tracking errors introduce a small change (1‐2 mm) to the penumbral width. When the penumbra is broad, particularly for non‐isocentric delivery and in the presence of tissue density heterogeneity, the dose coverage is more robust to translational errors. This suggests that the impact of tracking errors may be plan and patient specific.

### 4.2 Patient dose assessments

In a previous study,^12^ we evaluated the geometric impact of motion tracking errors and the dosimetric impact of uncorrected rotations for liver SBRT on CyberKnife. We recommended that the imaging isocenter be placed as close as possible to the center of fiducials assuming that target and fiducial centroids corresponded to each other. Although not directly addressed in this study, it is clear that minimizing the distance between fiducials and the targets is very important in an organ as deformable as the liver. In this larger dosimetric study, we combine motion tracking errors, uncorrected rotations, and a simple model of target deformation. Although several factors contribute to a lower dose coverage (including the standard deviation of the motion tracking errors), it is shown that large distances between the *target* and imaging isocenter combined with large rotations can lead to large reductions in target coverage. Similar findings have been reported for lung tumors treated on conventional linacs.^13^, ^14^ Treatment courses which are planned with higher D99/Rx are also shown to be less sensitive to uncorrected rotations and motion tracking errors.

Using two case studies, we illustrate how treatment plans can be made more robust to delivery errors by placing the imaging isocenter close as possible to the target centroid(s) during planning, and increasing planned D99/Rx for GTVs. Case 2 also demonstrates that target coverage can be improved if multiple distant targets are treated separately and not combined in the same plan. Since it is often known prior to treatment that it will not be possible to track rotations, another clinical application of our tool would be to assess the robustness of a plan against simulated rotation *prior* to treatment. In addition, our tool can provide an independent dosimetric assessment for treated fractions which can be used for inter‐fractional dose adaptation.

## 5 CONCLUSIONS

We introduce a dose perturbation tool validated under nine different experimental conditions. It accounts for translational and rotational motion‐tracking residual errors for homogenous and heterogeneous phantoms and in‐phase and out‐of‐phase motions across a wide range of phase shifts. The gamma analyses and comparisons of penumbral widths validate the model's performance at the 2%/2 mm level. This study also retrospectively assesses the dosimetric impact of delivery errors for patient's liver treatments and demonstrates how to improve the robustness of treatment plans to errors.

## AUTHOR CONTRIBUTIONS

Ming Liu: Study conception, data analysis, software development, and manuscript preparation. Joanna Cygler: Study conception, co‐supervision of the project and critical review of the manuscript. Kristopher Dennis: Study interpretation and critical review of the manuscript. Eric Vandervoort: Study conception and co‐supervision of the project, software development, critical review of the manuscript.

## CONFLICT OF INTEREST

The authors declare no conflict of interest.

## ETHICS APPROVAL

All data were collected with the informed consent of patients as a part of a Research Ethics Board approved local clinical trial (protocol number 20160594‐01H).
